# Concordance between European and US case definitions of healthcare-associated infections

**DOI:** 10.1186/2047-2994-1-28

**Published:** 2012-08-02

**Authors:** Sonja Hansen, Dorit Sohr, Christine Geffers, Pascal Astagneau, Alexander Blacky, Walter Koller, Ingrid Morales, Maria Luisa Moro, Mercedes Palomar, Emese Szilagyi, Carl Suetens, Petra Gastmeier

**Affiliations:** 1 Institute for Hygiene and Environmental Medicine, Charité – University Medicine Berlin, Campus Benjamin Franklin, Hindenburgdamm 27, D-12203, Berlin, Germany; 2 C-CLIN Nord - Département de santé publique, Université Pierre & Marie Curie, Paris, France; 3 Clinical Institute for Hygiene and Medical Microbiology, Medical University of Vienna, Vienna, Austria; 4 National Surveillance of Infections in Hospitals - NSIH, Operational Direction Public Health and Surveillance, Scientific Institute of Public Health, Brussels, Belgium; 5 Agenzia Sanitaria e Sociale Regione Emilia Romagna, Area di Programma Rischio Infettivo, Bologna, Italy; 6 Department of Intensive Care, Hospital Vall d'Hebron, Barcelona, Spain; 7 National Centre for Epidemiology, Department of Hospital Epidemiology, Budapest, Hungary; 8 European Centre for Disease Prevention and Control, Stockholm, Sweden

**Keywords:** Bloodstream infection, Pneumonia, Definitions, Healthcare-associated infections

## Abstract

**Background:**

Surveillance of healthcare-associated infections (HAI) is a valuable measure to decrease infection rates. Across Europe, inter-country comparisons of HAI rates seem limited because some countries use US definitions from the US Centers for Disease Control and Prevention (CDC/NHSN) while other countries use European definitions from the Hospitals in Europe Link for Infection Control through Surveillance (HELICS/IPSE) project. In this study, we analyzed the concordance between US and European definitions of HAI.

**Methods:**

An international working group of experts from seven European countries was set up to identify differences between US and European definitions and then conduct surveillance using both sets of definitions during a three-month period (March 1^st^ -May 31^st^, 2010). Concordance between case definitions was estimated with Cohen’s kappa statistic (κ).

**Results:**

Differences in HAI definitions were found for bloodstream infection (BSI), pneumonia (PN), urinary tract infection (UTI) and the two key terms “intensive care unit (ICU)-acquired infection” and “mechanical ventilation”. Concordance was analyzed for these definitions and key terms with the exception of UTI. Surveillance was performed in 47 ICUs and 6,506 patients were assessed. One hundred and eighty PN and 123 BSI cases were identified. When all PN cases were considered, concordance for PN was κ = 0.99 [CI 95%: 0.98-1.00]. When PN cases were divided into subgroups, concordance was κ = 0.90 (CI 95%: 0.86-0.94) for clinically defined PN and κ = 0.72 (CI 95%: 0.63-0.82) for microbiologically defined PN. Concordance for BSI was κ = 0.73 [CI 95%: 0.66-0.80]. However, BSI cases secondary to another infection site (42% of all BSI cases) are excluded when using US definitions and concordance for BSI was κ = 1.00 when only primary BSI cases, i.e. Europe-defined BSI with ”catheter” or “unknown” origin and US-defined laboratory-confirmed BSI (LCBI), were considered.

**Conclusions:**

Our study showed an excellent concordance between US and European definitions of PN and primary BSI. PN and primary BSI rates of countries using either US or European definitions can be compared if the points highlighted in this study are taken into account.

## Background

Implementation of surveillance of healthcare-associated infections (HAI) has been shown to result in decreasing HAI rates and contributes to the prevention of HAI [1-3]. Feedback of data on HAI rates to clinical staff has been shown to be a key factor reducing these rates [4-6].

Comparing HAI rates of one’s own institution with reference data seems to be particularly successful. In the 1970s, the US Centers for Disease Control and Prevention (CDC) created the National Nosocomial Infection Surveillance System (NNIS) and published uniform surveillance definitions for nosocomial infections [7-9]. These definitions have been updated gradually for surgical site infection (SSI) [10], for ventilator-associated pneumonia (VAP) [11], primary bloodstream infection (BSI) [12] and in 2010 for urinary tract infection (UTI) [13]. Key terms such as “device-associated infection” or “intensive care unit (ICU)-associated infection” were also defined [14]. This system is now integrated as part of the National Healthcare Safety Network (NHSN) [12].

In the 1980s and 1990s, many European countries performed national prevalence studies of HAI and established national surveillance systems using CDC definitions or a modified version of these definitions [15], while other countries developed their own surveillance definitions that better reflected European diagnostic practices. The first harmonization of national surveillance activities in Europe was performed by the Hospitals in Europe Link for Infection Control through Surveillance (HELICS) project, which was funded by the European Commission in the context of Decision 2119/98/EC of the European Parliament and of the Council on communicable disease surveillance and control in EU Member States [16].

The HELICS project (2000–2004) developed case definitions for surgical site infection (SSI), pneumonia (PN), bloodstream infection (BSI), catheter-related infection (CRI) and urinary tract infection (UTI) and recommended their use in EU Member States [17,18]. The work of HELICS was continued as a component of the European Commission-funded Improving Patient Safety in Europe (IPSE) network (2005–2008). The IPSE network aimed at contributing to European surveillance of HAI by describing HAI epidemiology, improving the understanding of inter-country variation of HAI rates and facilitating quality-of-care improvements in a multi-centre setting. In July 2008, the IPSE network was transferred to the European Centre for Disease Prevention and Control (ECDC) [19,20]. Since this date, HAI surveillance activities in Europe are coordinated by ECDC and the network was re-named the Healthcare-Associated Infections surveillance Network (HAI-Net). HAI-Net adopted the European (HELICS/IPSE) definitions for its HAI surveillance modules and for the ECDC point prevalence survey of HAI in European acute care hospitals. Comparisons of HAI rates between countries are essential to raising awareness about HAI and their prevention and control, but require a standardized methodology, including uniform definitions. Because they were implemented independently, national HAI surveillance systems in European countries decided to use either the US (CDC/NHSN) definitions [12-14] or the European (HELICS/IPSE) definitions [17,18] and questions have been raised about whether comparisons of HAI rates between national networks were indeed appropriate. While adoption of the European definitions is mandatory for newly implemented national HAI surveillance systems, changing definitions could interrupt continuity of reference data and require reorganization for an existing national surveillance system.

The present study was conducted to assess the concordance between US (CDC/NHSN) definitions and European (HELICS/IPSE) definitions of HAI for inter-country comparison of HAI rates. The study was initiated and sponsored by ECDC through a specific service contract (ECD.1781) with the Institute for Hygiene and Environmental Medicine, Charité – University Medicine Berlin, Germany.

## Methods

### Setting

The study was conducted in seven European countries (Austria, Belgium, France, Germany, Hungary, Italy and Spain) with existing networks for HAI surveillance. Network leaders and a senior expert from ECDC (CS) represented the international study working group responsible for the development and implementation of the study. Three meetings at the Institute of Hygiene, Charité – University Medicine, Berlin, were held to agree on the methodology, data collection and analysis.

A one-month study pre-test was performed in two countries to evaluate the feasibility of the study.

### Surveillance

HAI surveillance was performed in intensive care units (ICUs) in all participating countries between March 1^st^ and May 31^st^, 2010. Network leaders delivered study documents and trained the local surveillance personnel for both types of definitions by using standardized case studies. No validation phase was included in the study.

All patients aged one year or above that presented with symptoms for selected HAI were included in surveillance according to both types of definitions. The HAI did not necessarily need to be acquired in the participating ICU, and patients coming from another ward of the same hospital with symptoms of infection were also surveyed upon ICU admission.

Local surveillance personnel collected data by using both types of definitions simultaneously. A study case was defined as a patient with a HAI according to either type of definition. In addition to the definitions’ criteria, the following data were obtained for further analysis: date of birth, date of admission to the ICU and to the hospital, date of onset of HAI, underlying cardiac or pulmonary diseases, and immunosuppression status. In addition, surveillance personnel assessed whether the infection was ICU-acquired, according to both sets of criteria. Association with a central line or with mechanical ventilation was also included according to the US and the European definitions. BSI cases that did not fulfill the criteria of US definitions for laboratory-confirmed bloodstream infection (LCBI) because signs and symptoms were related to an infection at another site, were recorded as “secondary BSI missed by US definitions”.

### Statistical analysis

Because the question remains open as to which set of definitions represents the gold standard, we could only assess, for each type of infection, the concordance (agreement) between the two types of definitions. To estimate the concordance between two case definitions, Cohen’s kappa ( κ) statistic [21,22] was chosen.

For sample size calculation, an incidence of HAI (BSI and PN) of 1–5 per 100 patients was assumed and a kappa value of 0.75-0.90 was anticipated according to previous HAI concordance studies [23-26]. Based on a kappa value of 0.75 and on an expected HAI incidence of 1%, we estimated a sample size of 98 cases per infection type.

## Results

### Differences in definitions

Both sets of definitions of HAI were reviewed by the working group. The group identified differences for the definitions of BSI, PN and UTI (Table 1).

**Table 1:** Differences in HAI definitions (CDC/NHSN vs. HELICS/IPSE)

Type of HAI or key term	CDC/NHSN definitions	HELICS/IPSE definitions
Bloodstream infection (BSI) / Laboratory-confirmed bloodstream Infection (LCBI)	·LCBI (Positive blood culture with recognized pathogen or 2 blood cultures with skin contaminant incl. clinical symptoms. Organism cultured from blood is not related to an infection at another site)	·BSI-A
		(Positive blood culture with recognized pathogen or 2 blood culture with skin contaminant incl. clinical symptoms. Origin: “Catheter” (C), “Secondary to another site” (S) or “Unknown” (U))
	·CSEP (Clinical sepsis in patients ≤ 1 year)	
Catheter-related infection (CRI)	-*	·CRI 1 (Local central venous catheter (CVC)-related infection)
		·CRI 2 (General CVC-related infection)
		· CRI 3 (CVC-related BSI)
		· CCO (Catheter colonisation)
Pneumonia (PNU/PN)	· PNU1 (Clinically defined pneumonia)	· PN 1 (Protected sample + quantitative culture)
	· PNU2 (Pneumonia with specific laboratory findings)	· PN 2 (Non-protected sample + quantitative culture)
	· PNU3 (Pneumonia in immunocompromised patients)	· PN 3 (Alternative microbiological criteria)
		· PN 4 (Sputum bacteriology or non-quantitative endotracheal aspirate (ETA))
		· PN 5 (No microbiological criterion (only clinical criteria))
Urinary tract infection (UTI)	· SUTI (Symptomatic UTI) †/‡	· UTI-A (Symptomatic, microbiologically confirmed)
	· ASB (Asymptomatic bacteriuria) † /	
	·ABUTI (Asymptomatic bacteremic UTI) ‡	· UTI-B (Symptomatic , not microbiologically confirmed)
	· OUTI (Other infections of the urinary tract) †/‡	· UTI-C (Asymptomatic bacteriuria)
ICU-acquired HAI	· No evidence that the infection was present or incubating at the time of admission to the ICU	· Infection occurred later than 48 hours after admission in the ICU
Ventilator-associated	· A device to assist or control respiration continuously through a tracheostomy or by endotracheal intubation was present within the 48-hour period before the onset of infection, inclusive of the weaning period	· An invasive respiratory device was present (even intermittently) in the 48 hours preceding the onset of infection

*, not applicable.

†, until December 2008.

‡, since January 2009.

BSI definitions varied since CDC/NHSN does not accept a positive blood culture with a microorganism related to an infection at another site. PN definitions were different concerning the microbiological diagnostic criteria: HELICS/IPSE includes more detailed categories according to the sampling procedure and the microbiology technique whereas CDC/NHSN definitions include additional age-dependent criteria and a specific subcategory for immunocompromised patients (PNU3). UTI definitions were identical until the end of 2009. Differences appeared when CDC/NHSN modified its UTI definitions to include the new subcategory “asymptomatic bacteremic UTI” in January 2010.

Definitions of the key term “ICU-acquired infection” varied because HELICS/IPSE defines it as an infection occurring later than 48 hours after admission to an ICU, whereas CDC/NHSN requires that there is no evidence that the infection was present or incubating at the time of admission to the ICU, without time restriction [12]. There were also differences for the key term “mechanical ventilation”, which are described in Table 1.

The working group agreed to analyze concordance for the definitions of BSI and PN, and for the key terms “ICU-acquired infection” and “mechanical ventilation”. Since US and European definitions of UTI showed major differences because of recent modifications, UTI definitions were excluded from the study.

### Participating ICUs

Surveillance was performed in 47 ICUs in 28 hospitals across 7 EU countries. The majority of participating ICUs were mixed ICUs, followed by medical and surgical ICUs. Three countries also surveyed paediatric patients in 9% of their participating ICUs. The characteristics of participating ICUs are presented in Table 2.

**Table 2:** Participating intensive care units (ICUs)

Country	Number of ICUs / Number of hospitals	Number of ICUs per specialty*					Median number of beds per hospital	Median number of beds per ICU	Median number of beds with a ventilator per ICU (n)	Number of included patients
		Mixed	Internal medicine	Surgery	Cardiac surgery	Other†				
Austria	7 / 1	3	3	0	0	1	2,137	8	8	132
Belgium	5 / 4	3	1	1	0	0	854	18	18	1,318
France	4 / 4	2	0	2	0	0	504	11	11	323
Germany	5 / 1	2	0	0	1	2	3,200	11	11	689
Hungary	15 / 10	7	1	2	2	3	1,163	10	10	2,311
Italy	7 / 4	2	1	2	0	2	474	8	8	1,031
Spain	4 / 4	3	0	0	0	1	600	19	16	702
**All**	**47 / 28**	**22**	**6**	**7**	**3**	**9**	**854**	**11**	**11**	**6,506**

* An ICU was defined as belonging to a specialty if ≥ 80% of patients in this ICU belonged to this specialty.

† Other: Neurosurgery, Paediatrics, Transplant surgery, Burn, Neurology.

### Agreement of definitions

**Figure 1:**
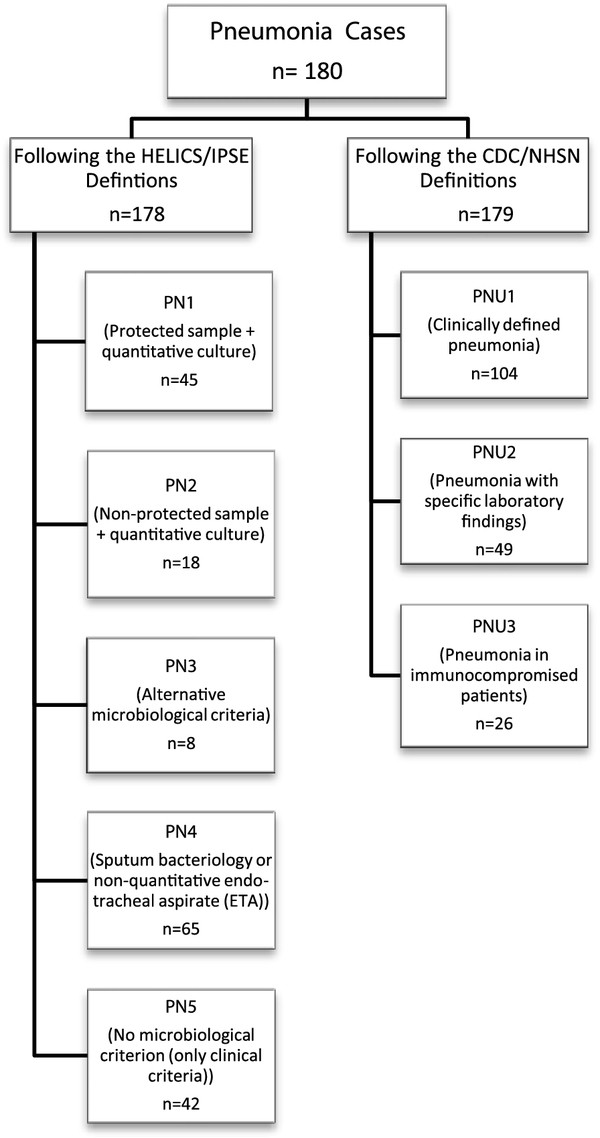
Pneumonia cases diagnosed according to both definition types.

**Figure 2:**
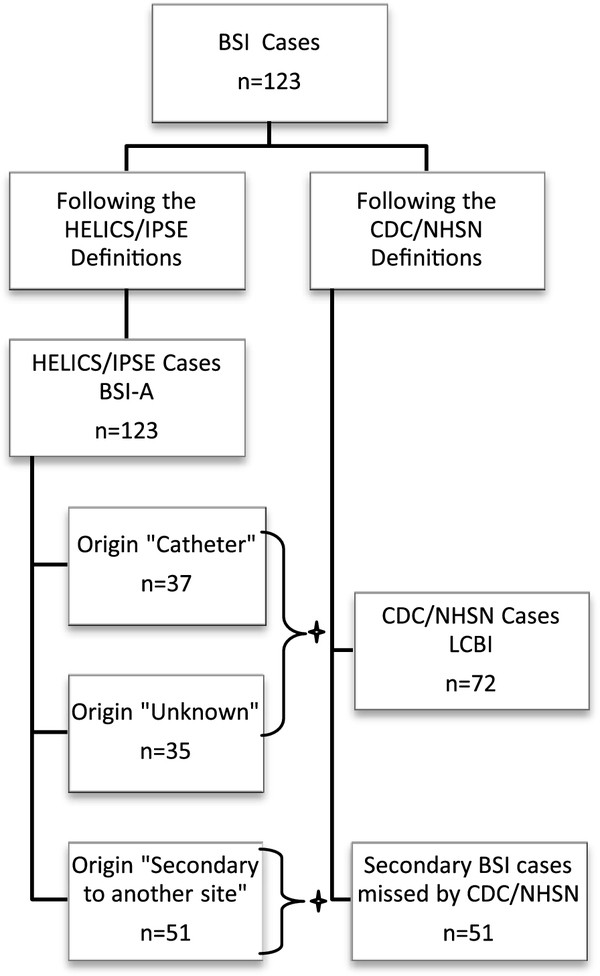
**BSI cases diagnosed according to both definition types, inclusive the mapping of conform criteria (+).**

For the study, 6,506 patients were assessed. The incidence of PN and of BSI were 2.8 and 1.9 per 100 patients, respectively. Overall, 180 PN and 123 BSI cases were identified by either the US definitions or the European definitions (Figures 1 and 2). Of all 180 PN cases, 178 were identified with the European definitions and 179 with the US definitions. Two PN cases were only identified with the US definitions due to age-dependent criteria that are not included in the European definitions. The third discordant case was a patient with microbiological findings that fulfilled a criterion for “PN 2” of the European definitions, but without sufficient criteria for PN according to the US definitions. These findings led to a kappa value of 0.99 for PN. Kappa values were lower when PN cases were subdivided into clinically defined PN (κ = 0.90) and microbiologically defined PN (κ = 0.72).

Since this subdivision did not take into account US-defined PNU3 cases (PN in immunocompromised patients), those cases were reclassified into US-defined PNU1 and PNU2. A repeated analysis of agreement within the new clinically and microbiologically defined PN groups resulted in equal agreement for clinically defined PN (κ = 0.90) and higher agreement for microbiologically defined PN (κ = 0.84).

Agreement of definitions for BSI showed a kappa value of 0.73. All 123 BSI cases were diagnosed by the European criteria. Forty-two percent of the BSI cases were missed when US definitions were used (Figure 2) because they were secondary to an infection at another site. In the remaining 72 cases, the BSI origin was either a catheter (central venous, peripheral or arterial) (30%) or unknown (29%). BSI concordance was perfect (κ = 1.00) when only primary BSI cases, i.e. Europe-defined BSI with either “catheter” or “unknown” origin and US-defined “LCBI”, were analyzed.

For 245 (81%) of all cases the concordance of the key term “ICU-acquired” was analyzed. A few more HAI were classified as “ICU-acquired” according to the US definitions than to the European definitions (245 vs. 240); agreement was equal for ICU-acquired PN and for ICU-acquired BSI (κ = 0.94) (Table 3).

**Table 3:** Concordance of HAI definitions, determined by Cohen’s kappa statistic

Type of HAI or key term	Included cases based on:		Incidence of HAI (no. cases per 100 patients)	No. cases of HAI				No. patients without HAI	Cohen’s kappa [95% confidence interval]
	US definitions	European definitions		According to either the European or the US definition	According to both the European and the US definitions	According to the European definition but not the US definition	According to the US definition but not the European definition		
Pneumonia	PNU1 + PNU2 + PNU3	PN1 + PN2 + PN3 + PN4 + PN5	2.8	180	177	1	2	6,326	0.99 [0.98 ; 1.00]
Clinically defined pneumonia	PNU1	PN2 + PN4 + PN5	2.0	127	102	23	2	6,379	0.89 [0.85 ; 0.93]
	PNU1	PN4 + PN5	1.8	119	92	15	12	6,387	0.87 [0.82 ; 0.92]
	PNU1*	PN2 + PN4 + PN5	2.0	127	104	21	2	6,379	0.90 [0.86 ; 0.94]
	PNU1*	PN4 + PN5	1.8	119	94	13	12	6,387	0.88 [0.83 ; 0.93]
Microbiologically defined pneumonia	PNU2	PN1 + PN3	1.0	65	37	16	12	6,441	0.72 [0.63 ; 0.82]
	PNU2	PN1 + PN2 + PN3	1.2	78	42	29	7	6,428	0.70 [0.60 ; 0.79]
	PNU2†	PN1 + PN3	1.1	73	53	0	20	6,433	0.84 [0.77 ; 0.91]
	PNU2†	PN1 + PN2 + PN3	1.3	84	60	11	13	6,422	0.83 [0.77 ; 0,90]
ICU-acquired pneumonia	Pneumonia not present or in incubation at admission	Pneumonia occurring >48 h after admission	2.3	147	144	0	3	28	0.94 [0.87 ; 1.00]
Mechanical ventilation	Continuous presence of device within 48 hours preceding pneumonia onset	Presence of device (even intermittently) within 48 hours preceding pneumonia onset	2.1	136	134	2	0	42	0.97 [0.93 ; 1.00]
Bloodstream infection (BSI)	Microorganism is not related to infection at another site	Origin of BSI is “catheter”, “secondary to another site” or “unknown”	1.9	123	72	51	0	6,383	0.73 [0.66 ; 0.80]
Primary BSI	Microorganism is not related to infection at another site	Origin of BSI is “catheter” or “unknown”	1.1	72	72	0	0	51	1.00
ICU-acquired BSI	BSI not present or in incubation at admission	BSI occurring >48 h after admission	1.5	98	96	0	2	22	0.94 [0.87 ; 1.00]

*Including PNU3 cases (pneumonia in the immunocompromised patient) qualified as PNU1 after redistribution of PNU3 cases into PNU1 or PNU2.

† Including PNU3 cases (pneumonia in the immunocompromised patient) qualified as PNU2 after redistribution of PNU3 cases into PNU1 or PNU2.

## Discussion

HAI surveillance methods vary across Europe. Some countries use European definitions while other countries use US definitions. As a contribution to further harmonization of Europe-wide surveillance of HAI, this study assessed the concordance between US and European definitions of BSI and of PN, two major types of HAI that are partly preventable [27] and are under surveillance in most European countries. The recommendations of Landis and Koch for evaluating the strength of an agreement were used [28]. Overall, an “almost perfect” agreement was found for PN (κ = 0.99). This was different when PN cases were subdivided into clinically and microbiologically defined PN. More PN cases were classified as microbiologically-defined PN following the European definitions than the US definitions. This was still the case when Europe-defined PN2 cases which are based on the criteria “non-protective sample and quantitative culture” were considered as clinically defined PN. This difference was no longer evident when all US-defined PNU3 (“PN in immunocompromised patients”) cases were reclassified into the US-defined categories PNU1 or PNU2. Since all 26 PNU3 cases could be classified as either PNU1 cases (n = 2) or PNU2 cases (n = 24), the results of this study suggest that the PNU3 subcategory may not be essential when performing surveillance of PN in immunocompromised patients.

As expected, concordance of BSI definitions was only “substantial” according to Landis and Koch [28]. Since one major criterion of US definitions, i.e. signs and symptoms of BSI must not be related to an infection at another site, is not included in the European definitions, 51 (42%) BSI cases were not identified with the US definition. With the European BSI definition, which includes the specification of the origin of the BSI, these 51 BSI cases were reported as “secondary to another infection site”.

European definitions provide two more categories, i.e. “catheter” and “unknown”, for origin of BSI [18]. The origin “catheter” was reported in 37 (30%) BSI cases and the origin “unknown” was reported in 35 (29%) BSI cases, which correspond to the “primary BSI” of the US definitions. All 72 of these BSI cases were also defined as LCBI cases with the US definitions. Thus for a potential comparison, US-defined LCBI cases should only be related to Europe-defined BSI cases with either “catheter” or “unknown” origin.

Definitions of the key term “ICU-acquired HAI” varied between the U.S. and Europe. According to European definitions 97% of HAI cases were defined as ICU-acquired (i.e. HAI occurring later than 48 hours after admission in the ICU). By contrast, according to the US definitions, all HAI cases were defined as ICU-acquired (i.e., no evidence that the infection was present or incubating at the time of admission to the ICU). Since US definitions do not specify a time period between admission to the ICU and onset of symptoms, it is easy to explain why a few more HAI were recorded as ICU-acquired according to the US definitions. Nevertheless, agreement for the key term “ICU-acquired HAI” was still “almost perfect” according to Landis and Koch [28].

A strength of our study is that it was performed in seven European countries with different diagnostic methods and habits reflecting the variety of diagnostic practices in Europe. A limitation of the study is that the time of admission to the ICU and the time of onset of the HAI were recorded less precisely (in “days” instead of “hours”) than in the original definition because the findings of the study pre-test revealed major difficulties in collecting time of onset data in “hours”. As a consequence, all HAI occurring on or after the third day of ICU stay (rather than after 48 hours according to the European definitions), were defined as ICU-acquired. A further limitation was that specific patient groups, such as paediatric and immunocompromised patients, were likely to be underrepresented in the study.

In conclusion, countries using either US or European definitions for HAI surveillance can compare PN and primary BSI rates as long as the following points are taken into account. First, data should of course be valid and be collected following the original US and European definitions since country-specific modification of definitions may result in additional differences [29]. Second, PN data should always be compared in total, but not as subcategories of clinically defined and microbiologically defined PN. Third, for BSI the source should always be reported since all BSI cases with the origin “secondary to another site” according to European definitions should be excluded when making comparisons with US-defined BSI. Fourth, only Europe-defined BSI cases with either “catheter” or “unknown” origin should be compared to US-defined LCBI cases. Fifth, there are differences between US and European surveillance protocols, other than just case definitions of HAI, and these differences should be taken into account before performing comparison of HAI rates. Finally, comparisons are valid as long as US and European definitions do not change. Indeed, changing US definitions for PN to ventilator-associated complications under the influence of public reporting [30,31] would certainly affect the current good concordance between US and European definitions of PN.

## Abbreviations

BSI: Bloodstream infection; CDC: Centers for disease control and prevention; ECDC: European Centre For Disease Prevention And Control; HAI: Healthcare-associated infection; HELICS: Hospitals in Europe Link for Infection Control through Surveillance; ICU: Intensive care unit; IPSE: Improving patient safety in Europe; k: Kappa; LCBI: Laboratory-confirmed bloodstream infection; NHSN: National Healthcare Safety Network; NNIS: System National Nosocomial Infection Surveillance System; PN: Pneumonia; PN1-5: Pneumonia case definitions (according to HELICS/IPSE); PNU1: Clinically defined pneumonia (according to CDC/NHSN); PNU2: Pneumonia with specific laboratory findings (according to CDC/NHSN); PNU3: Pneumonia in immunocompromised patients (according to CDC/NHSN); SSI: Surgical site infection; UTI: Urinary tract infection.

## Competing interests

The study was supported by the European Centre for Disease Prevention and Control (ECDC) through a specific service contract (ECD.1781). The authors declare that they have no competing interests.

## Authors’ contributions

The study was designed by all members of the working group (PA, AB, PG, SH, WK, IM, MLM, MP, ES, CS) and by CG and DS. PG was the investigator of the study. SH coordinated the study. PA, AB, SH, IM, MLM, MP, ES performed the study in their respective countries. DS performed the statistical analysis. SH drafted the manuscript. All authors critically revised the manuscript and contributed substantially to the submitted version. All authors have read and approved the final manuscript.
